# 2D Organic–Inorganic Hybrid Perovskite Quantum Well Materials and Their Dramatical X-ray Optoelectronic Properties

**DOI:** 10.3390/ma14195539

**Published:** 2021-09-24

**Authors:** Huiwen Chen, Yunlong Li, Dongfeng Xue

**Affiliations:** Multiscale Crystal Materials Research Center, Shenzhen Institute of Advanced Technology, Chinese Academy of Sciences, Shenzhen 518055, China; hw.chen1@siat.ac.cn

**Keywords:** X-ray, two-dimensional, quantum well, hybrid perovskite

## Abstract

Two-dimensional organic–inorganic hybrid perovskites (2D OIHPs) have attracted extensive attention in the field of X-ray detection due to their excellent stability compared to traditional three-dimensional OIHPs and the strong optoelectronic response to X-ray along the quantum wells. In this review, the nucleation and growth process as well as intermolecular forces for controlling out-of-plane growth are summarized along with the oriented growth mechanism. The optoelectronic properties in 2D OIHP under irradiation of X-ray are also discussed. Finally, conclusions and outlook for orientation 2D OIHP quantum wells and their challenges in application of direct X-ray detection are given. This review will provide a basic understanding on the strategy of designing 2D OIHP thick films as promising X-ray photoconductors, which may inspire the development of next-generation X-ray detectors.

## 1. Introduction

**Figure 1:**
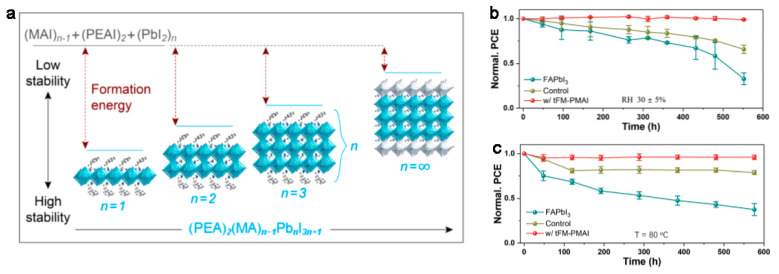
(**a**) The formation energy of (PEA)_2_(MA)*_n_*_−1_Pb*_n_* I_3*n*+1_ with different *n* values. Reprinted with permission from [10], Copyright 2016 American Chemical Society. (**b**) The ambient stability and (**c**) thermal stability of 3D perovskite FAPbI_3_ and 2D OIHP w/tFM-PMAI. Reprinted with permission from [9], Copyright 2016 John Wiley and Sons.

Recently, two-dimensional organic–inorganic hybrid perovskites (2D OIHPs) have aroused increasing attention owing to their superior operational stability with unencapsulated control compared to their 3D counterparts [1,2,3]. The main strategy for constructing 2D OIHPs is to introduce long chain organic cations in 3D OIHPs (general formula of ABX_3_) [4]. The introduction of organic spacers will increase the formation energy (shown in Figure 1a) and ion migration activation energy of 2D OIHPs, thereby successfully reducing the decomposition and phase transformation of perovskites [5,6,7]. In addition, the high hydrophobicity of the spacers can block the water molecules to increase the humidity and thermal stability of 2D OIHPs, as shown in Figure 1b,c [8,9].

**Figure 2:**
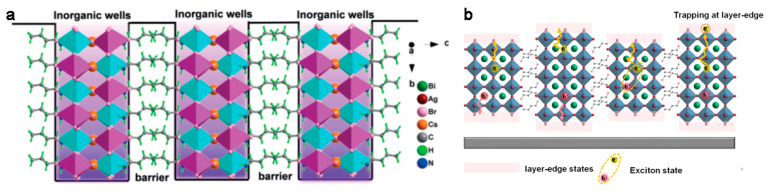
(**a**) Structural configuration of (BA)_2_CsAgBiBr_7_ that defines the 2D perovskite quantum-confined motif. Reprinted with permission from [12], Copyright 2019 John Wiley and Sons. (**b**) Schematics of the low energy state of layer edge in quantum well separating excitons into free carriers.

The typical formula of 2D OIHPs is A*_n_*_−1_A′B*_n_*X_3*n*+1_ (Dion-Jacobson, DJ) or A*_n_*_−1_A′_2_B*_n_*X_3*n*+1_ (Ruddlesden–Popper, RP), where the [BX_6_]^4−^ octahedron acts as a well, while the organic layer A′ functions as a barrier and *n* determines the thickness of the quantum well (Figure 2a) [10,11]. Generally, the carriers confined in the quantum wells lead to high exciton binding energy and enlarge the formation of excitons rather than free carriers, thereby limiting the carrier transport in 2D OIHPs [12,13]. However, the exciton binding energy can be decreased by tuning the *n* of the quantum well [14]. When *n* > 2, the low energy state of the layer edge in the quantum well will separate excitons into free carriers [15], which is beneficial for the electrical properties, as shown in the schematic drawing in Figure 2b.

In addition, the inhibition of charge transport between quantum wells is attributed to the small dielectric constant of organic spacers, which is unfavorable for efficient charge collection [16]. Changing the orientation of the quantum well to be perpendicular to the electrode will improve the crystallinity of the 2D OIHP and is an effective way to improve the performance of the corresponding optoelectronics as the carriers are confined in quantum wells [17].

In recent years, 2D OIHP quantum well has gradually been studied as a photoconductor in X-ray detectors, which can be functioned as a natural array material for pixel matrix thin film transistor (TFT). The charge carrier transport exhibits obvious anisotropy in 2D OIHPs that is strong along the growth direction of the octahedron and weak at the perpendicular direction. Therefore, in order to improve the X-ray optoelectronic properties of 2D OIHPs to obtain highly sensitive X-ray detectors, a thick film with vertically oriented 2D OIHP quantum well is necessary.

In this article, we focus on the nucleation and growth process as well as intermolecular forces for controlling out-of-plane growth and summarize the oriented growth mechanism of 2D OIHP quantum wells. We also discuss the optoelectronic properties in 2D OIHPs under irradiation of X-ray. Finally, conclusions and outlook for orientation 2D OIHP quantum wells and their challenges in application of direct X-ray detection are given. We believe this review will provide guidance for designing and fabricating promising X-ray photoconductors that could be used in next-generation X-ray imaging detectors.

## 2. Regulation of Out-of-Plane Oriented Growth

Controlling the orientation is an efficient way to improve the carrier transport of 2D OIHPs. There are three strategies to achieve vertical orientation growth of 2D OIHP quantum well on the substrate in situ: (i) regulating the nucleation process, (ii) controlling the growth process, (iii) altering the intermolecular forces of organic spacers.

### 2.1. Nucleation Process

#### 2.1.1. Solvent Engineering

**Figure 3:**
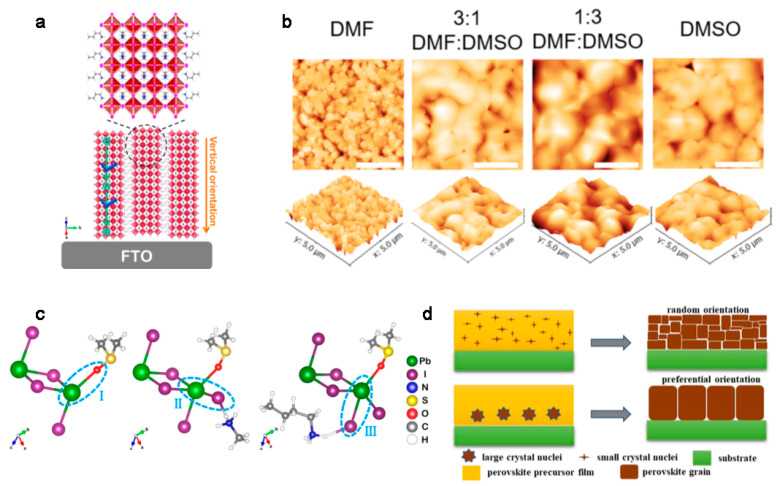
(**a**) Schematic of the horizontal and vertical orientations relative to FTO substrate. Reprinted with permission from [21], Copyright 2019 American Chemical Society. (**b**) AFM images of the hot-cast BA_2_MA_4_Pb_5_I_16_ films from precursor solutions using DMF, DMSO, and mixed solvents of the two, illustrating a reduction in surface roughness upon addition of DMSO to the DMF precursor solution. The RMS values of the DMF, 3:1 DMF:DMSO, 1:3 DMF:DMSO, and DMSO films are 28.9, 11.26, 14.35, and 10.6 nm, respectively. The size of each image is 5 × 5 µm^2^. Reprinted with permission from [23], Copyright 2017 John Wiley and Sons. (**c**) Sketch diagram for the interaction force between (i) PbI_2_and DMSO, (ii) PbI_2_and MAI, and (iii) PbI_2_ and BAI. Reprinted with permission from [21], Copyright 2019 American Chemical Society. (**d**) Illustration of the formation of perovskite film with lots of small crystal nuclei or with a small amount of large crystal nuclei. Reprinted with permission from [17], Copyright 2020 Elsevier.

Solvent engineering has been proven to be an effective way to control crystallization and orientation of 2D OIHPs. Solvents with different polarities, boiling points, and coordinating ability will affect the crystallization kinetics and thus change the orientation of 2D OIHPs [4,18,19]. DMSO is the most commonly used solvent additive to adjust the orientation perpendicular to the substrates in 2D OIHPs. With the addition of DMSO in 2D OIHPs dissolved in DMF, the 2D OIHP films tend to form vertical orientation to the substrate (Figure 3a) and result in increased crystallinity [14,20,21]. Simultaneously, the grain size dramatically increases when the DMSO ratio is increased (Figure 3b) [22].

The vertical orientation after addition of DMSO is attributed to the interaction between the Lewis acid Pb^2+^ and the Lewis base S=O in DMSO, which forms the intermediate product BAI/MAI/PbI_2_/DMSO (Figure 3c) and results in the formation of large crystal nuclei that slow the crystallization process. When only DMF is used, the precursor undergoes direct and fast volatilization, resulting in random orientation of 2D OIHP films (Figure 3d) [14,16,23].

#### 2.1.2. Additive Engineering

Additive engineering is another method to improve the orientation of 2D OIHP quantum wells. Generally, NH_4_X (X = Cl, Br, I, SCN [24,25,26,27]), thiourea (THA) [28], thiosemicarbazone (TSC) [14], etc. are used to control the orientation and improve the morphology of 2D OIHPs.

**Figure 4:**
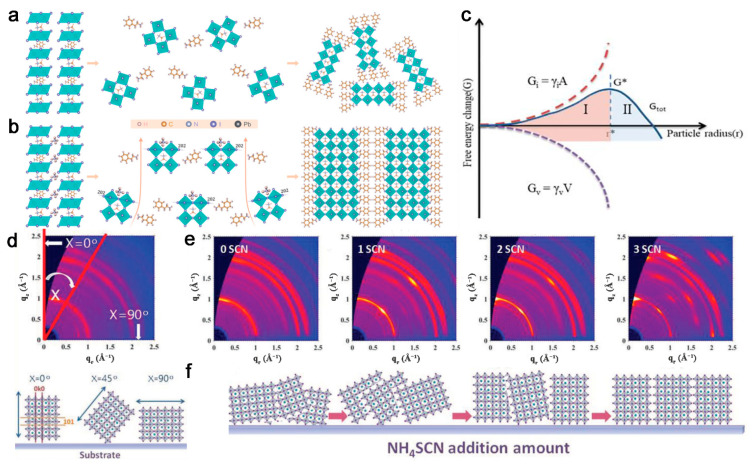
A schematic diagram of (**a**) the random crystallization of (PEA)_2_(MA)_3_Pb_4_I_13_ in the conventional method and (**b**) the vertical orientation showing the flattened (202) crystal plane caused by NH_4_Cl evaporation. Reprinted with permission from [31], Copyright 2020 RSC Publishing. (**c**) Dependence of free energy change on particle radius. Reprinted with permission from [17], Copyright 2020 Elsevier. (**d**) The schematic of azimuth angle (χ) evolution of (101) crystallographic. (**e**) 2D GIWAXS patterns of (BDA)(MA)_4_Pb_5_I_16_ (*n* = 5) perovskite films with 0–3 SCN^−^ with an incidence angle of 0.3°. (**f**) The schematic of the orientation evolution of (101) crystallographic plane that occurs with increasing NH_4_SCN addition. Reprinted with permission from [33], Copyright 2019 John Wiley and Sons.

NH_4_Cl can stick to the (202) surface and enhance the solubility of [BX_6_]^4−^ octahedral colloid by interaction between the Lewis base Cl^−^ and the Lewis acid Pb^2+^ or Sn^2+^ at the B site and inhibit homogenous nucleation inside the solution. The escape of NH_4_Cl during the annealing leaves the flat (202) planes, and the position perpendicular to these planes will be spontaneously occupied by the organic spacers to form the longitudinal growth of [BX_6_]^4−^, thereby leading to vertical orientation (Figure 4a,b) [19,29,30]. In addition, NH_4_Cl will reduce the diameter of the pinholes to form a uniform morphology [31].

With the increase in amount of NH_4_SCN additives, sharp and discrete Bragee spots are observed in GIWAXS patterns, revealing the preferential orientation of (101) from randomly orientated to parallel to the substrate (Figure 4c–e) [32]. NH_4_SCN can coordinate with Pb^2+^ by SCN^−^ in solution, and the NH_4_SCN will then evaporate during the following annealing period. This will enlarge the crystalline grains, decrease the grain boundaries, and improve the vertically oriented growth of 2D OIHP films [33].

THA and its derivative TSC play a similar role in optimizing orientation of 2D OIHPs [14,22]. TSC is a S-donor Lewis base containing –NH_2_, which not only strongly bonds with PbI_2_ but also interacts with organic ammonium salt in 2D OIHPs to form an intermediate with a size larger than the critical radius r* (region Ⅱ in Figure 4f). r* is the critical particle radius corresponding to the critical free energy G*, which should be overcome during the formation of nuclei. When the particle radius is larger than r*, the crystal grows spontaneously with no need to overcome G*, leading to a reduction in nucleation density and an increase in the crystallinity and vertically orientated crystal grains [14].

**Figure 5:**
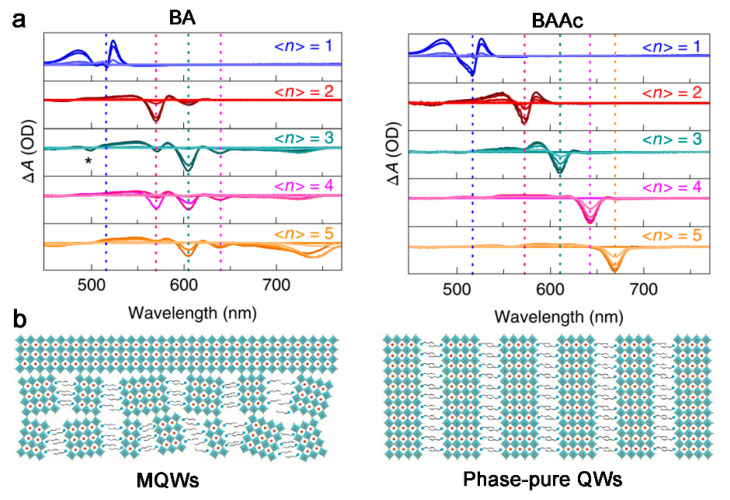
(**a**) TA spectra and (**b**) schematic structure of the multiquantum wells (MQW, with organic spacer BA) and phase-pure QW films (with organic spacer BAAc). Reprinted with permission from [34], Copyright 2020 Springer Nature.

Introducing polarity functional group into the organic spacers can also regulate the nucleation rate of 2D OIHPs. Liang et al. prepared a molten spacer butylamine acetate (BAAc) by injecting acetic acid (Ac) into n-butylamine (BA). In BAAc, Ac^−^ can form strong coordination with Pb^2+^ to obtain a uniformly distributed gel and prevent the aggregation of [PbI_6_] octahedron, thereby allowing phase-pure quantum wells, larger crystal grains, and superior orientation to the substrate formed in 2D OIHPs (Figure 5) [34].

#### 2.1.3. Composition Engineering

**Figure 6:**
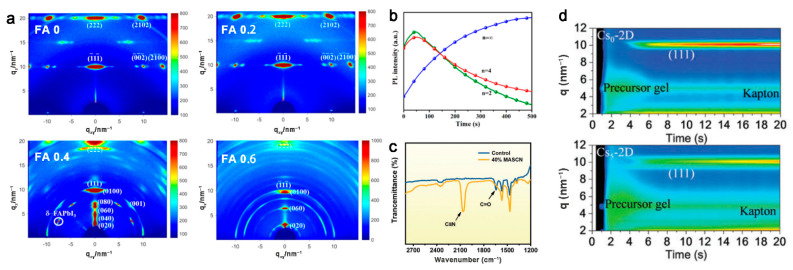
(**a**) GIWAXS patterns of the perovskite films of (BA)_2_(MA_1−x_FA_x_)_3_Pb_4_I_13_, *x* = 0, 0.2, 0.4, and 0.6 films. (**b**) In situ PL measurement for the BA_2_(MA)_3_Pb_4_I_13_ film by detecting the emission peak at 585 (*n* = 2), 670 (*n* = 4), and 780 (*n* = ∞) nm. Reprinted with permission from [35], Copyright 2018 American Chemical Society. (**c**) FTIR spectra of the control and 40% MASCN sample without annealing. Reprinted with permission from [37], Copyright 2021 John Wiley and Sons. (**d**) In situ GIWAXS patterns showing the phase transition from precursor to perovskite for the Cs0-2D and Cs5-2D samples. Reprinted with permission from [38], Copyright 2018 RSC Publishing.

Doping different cations on the A site in 2D OIHPs, e.g., BA_2_(MA_1−x_FA_x_)_3_Pb_4_I_13_, is an method employed to inhibit the generation of low *n* phase, which is unfavorable for vertical orientation to substrates (Figure 6a,b) [35,36].

Increasing the same cation on the A site in the 2D OIHP quantum wells has a preferred orientation mechanism that is completely different from the former one. Addition of MASCN in the precursor with DMF as solvent will suppress formation of the intermediate phase, i.e., [PbI_6_]^4−^ sol–gel, which will transform into an unoriented intermediate phase that tends towards random orientation, accompanied by the disappearance of the C=O signal of DMF (Figure 6c). It has been proven that excess MA^+^ can improve the orientation of 2D OIHP function as a shell dividing the [PbI_6_]^4−^ cages and preventing the assemble of [PbI_6_]^4−^ after excluding the function of SCN^−^ [37].

In addition, Cs^+^ doping on the A site will slow the crystallization kinetics, which will help the grains grow larger and retain perfect crystal orientation (Figure 6d) [38]. Furthermore, the formation of crystal nucleus in the early stage of spin-coating, attributed to much lower solubility of KI in DMF, will induce heterogeneous crystallization to achieve a quantum well perpendicular to the substrate [39].

### 2.2. Growth Process

#### 2.2.1. Temperature

**Figure 7:**
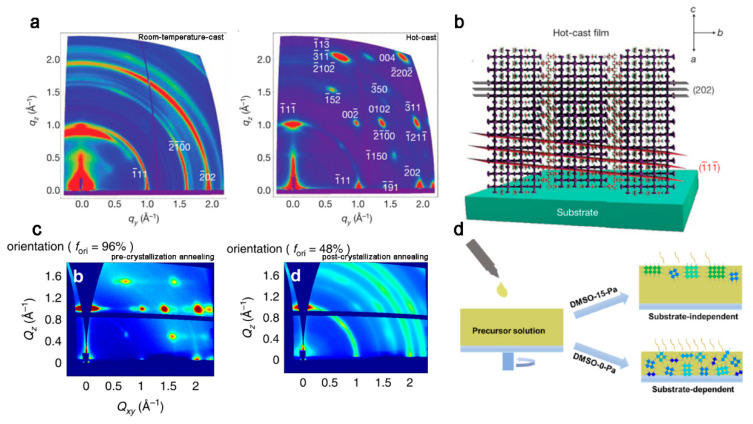
(**a**) GIWAXS patterns for polycrystalline room-temperature-cast and hot-cast (BA)_2_(MA)_3_Pb_4_I_13_ films. (**b**) Schematic representation of the (101) orientation, along with the (111) and (202) planes of a 2D perovskite crystal, consistent with the GIWAXS data. Reprinted with permission from [40], Copyright 2016 Springer Nature. (**c**) GIWAXS patterns for pre- and postcrystallization annealed films. Reprinted with permission from [45], Copyright 2018 Springer Nature. (**d**) Schematic of the in situ crystallization modes of the perovskites. Reprinted with permission from [46], Copyright 2021 American Chemical Society.

Tsai et.al. first used hot-casting to obtain vertical orientation (BA)_2_(MA)_3_Pb_4_I_13_ films, which yielded sharp Bragg spots in the GIWAXS patterns and was oriented with (101) planes (Figure 7a,b) [40]. It is believed there is accelerated conversion of the intermediate to 2D OIHP during the hot-casting process [41].

In previous studies, annealing can also influence the vertical orientation [42,43,44]. Chen, et.al. operated the annealing process before and after the crystalline of 2D OIHP films, named as pre- and postcrystallization annealing methods. The precrystallization annealed film showed superior vertical orientation compared to the postcrystallization annealed ones. This is because precrystallization annealing keeps the high evaporation rate of the solvent, while postcrystallization annealing decreases the diffusion rates of the precursor, thereby causing formation of homogeneous nucleation and randomly oriented films (Figure 7c) [45].

#### 2.2.2. Vapor Pressure

Controlling the solvent vapor pressure will result in different crystallization modes in perovskite. With DMSO partial pressure of 15 Pa, the solvent evaporation slows down, resulting in crystallization at the air–liquid interface. Meanwhile, at partial pressure of 0 Pa, the fast evaporation of the solvent leads to simultaneous crystallization of the liquid–solid and air–liquid interfaces (Figure 7d) [46]. This phenomenon is similar to the result of annealing at different stages of crystallization. Although controlling vapor pressure has not been used in the oriented growth of 2D OIHP quantum well, we believe it will be an effective strategy.

### 2.3. Intermolecular Forces of Organic Spacers

**Figure 8:**
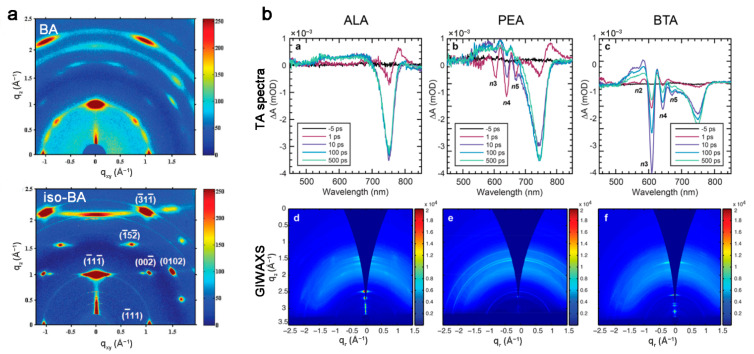
(**a**) GIWAXS patterns of (BA)_2_(MA)_3_Pb_4_I_13_ and (iso-BA)_2_(MA)_3_Pb_4_I_13_ perovskites. Reprinted with permission from [47], Copyright 2017 John Wiley and Sons. (**b**) TA spectra and GIWAXS patterns of ALA-, PEA-, and BTA (i.e., BA)-based 2D OIHP films. Reprinted with permission from [49], Copyright 2018 American Chemical Society.

Various organic spacers (A′) are employed to construct 2D OIHP quantum wells, and their intermolecular force is an important factor to tune the orientation of 2D OIHP. The rigid benzene ring in PEA may confine the freedom compared to BA, leading to a favorable oriented structure [24]. Shortening the chain length of spacers can also improve the orientation of 2D OIHP, e.g., 2D OIHP prepared by inserting the branched-chain butylamine (iso-BA) into anionic layers displays sharper Bragg spots than that prepared by BA, illustrating dramatically enhanced orientation (Figure 8a) [47]. Because allylammonium (ALA) has only the single C=C bond to promote intermolecular force, there is lower formation energy of low *n* quantum wells. PEA and BA will form intermolecular forces by π-stacking and van der Waals interaction, leading to higher formation energy of low *n* quantum wells. Thus, ALA-based 2D OIHP have higher degree of orientation and narrower distribution of quantum wells width than PEA- and BA-based ones (Figure 8b) [48,49]. The stronger interaction between sulfur atoms in two 2-(methylthio)-ethylamine hydrochloride (MTEA) also enables more oriented growth than weak van der Waals interaction between BA [50].

**Figure 9:**
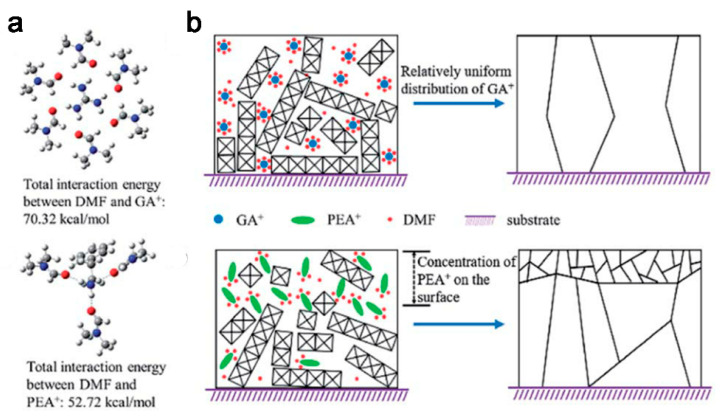
(**a**) The schematics of the solvation shell of GA^+^ and PEA^+^ in DMF and (**b**) the influence of GA^+^ and PEA^+^ on the formation of 2D perovskite film. Reprinted with permission from [53], Copyright 2019 RSC Publishing.

Introducing binary organic spacers can successfully build fabricated vertically oriented structure as the second spacer can generate aggregates that tend to form large crystal grains with preferable vertical orientation [51,52]. Inserting structurally symmetric guanidinium (GA) as a second spacer in the BA_2_MA_4_Pb_5_I_16_ 2D OIHP film can result in better crystallinity than using PEA because GA has better solubility in the solvent DMF, thereby allowing even distribution of amino groups and favoring the formation of oriented structure (Figure 9) [53].

## 3. Oriented Growth Mechanism

**Figure 10:**
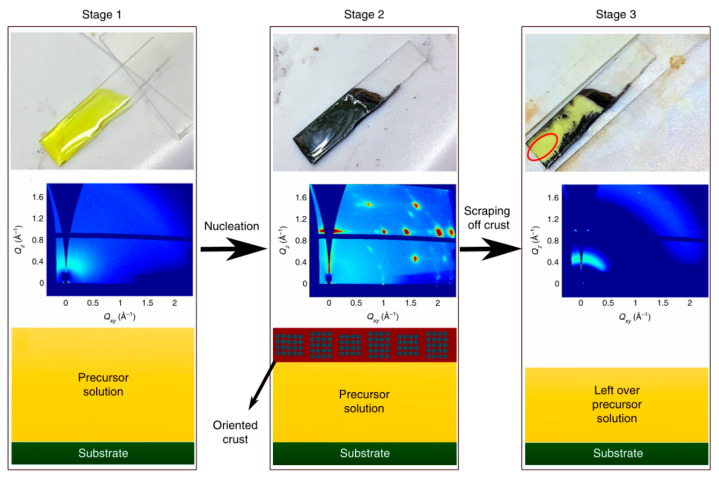
Optical images, GIWAXS patterns, and illustrations of precursor solution (**stage 1**), BA_2_MA_3_Pb_4_I_13_ top-crust crystallization (**stage 2**) and scraping off crust (**stage 3**). Reprinted with permission from [45], Copyright 2018 Springer Nature.

The vertical self-assembly process of 2D OIHP remains a mystery, which limits the control of vertical orientation. Chen et.al. removed the top crust of the film using a home-made blade setup and performed in situ GIWAXS characterization to these products (Figure 10). The results confirm that vertically oriented nucleation occurs from the liquid–air interface because the surface tension makes the BA molecule staying inside the solution bound to [PbI_6_] slabs and form initial nuclei vertical to the substrate at the liquid–air interface. Then, BA_2_MA_3_Pb_4_I_13_ crystallizes from the nuclei during the annealing process [45].

However, the rule is not always suitable for other systems, e.g., increasing the volatilization rate of solvent by reducing the partial pressure will cause a synchronous crystallization at both the liquid–air surface and solid–liquid surface as discussed above [46].

## 4. X-ray Optoelectronic Properties

2D OIHPs have already emerged as promising materials in the field of photovoltaics due to their tunability of optoelectronic properties and, more importantly, environmental stability. Recently, tremendous effort has been dedicated to 2D perovskites to realize X-ray detection because of their quantum well (QW) structures, where the semiconducting inorganic sheets act as the wells and the organic dielectric layers correspond to the barriers. This unique character makes 2D OIHPs natural array materials for pixel thin film transistor (TFT) substrates, which indicates great potential in direct X-ray imaging.

Wang et al. obtained a large (BDA)PbI_4_ single crystal by cooling down the solution slowly. The 2D OIHP achieved X-ray sensitivity of up to 188 μC Gy_air_^−1^ cm^−2^ at 50 V voltage under irradiation by an X-ray tube with acceleration voltage of 40 kV (40 kVp, soft X-rays). The value was 9-fold higher than the dominant commercial X-ray detector a-Se (20 μC Gy_air_^−1^ cm^−2^). The lowest detection limit of (BDA)PbI_4_ was 1.58–3.13 µGy_air_·s^−1^ at 10–50 V voltage bias, which is lower than the regular medical diagnostics and makes 2D (BDA)PbI_4_ a candidate material for soft X-ray detector [54].

For hard X-rays, 2D OIHPs outperform the dominating CsI scintillator of commercial digital radiography systems by acquiring clear X-ray images under much lower dose rate. Wei et al. introduced deficient F atoms to enhance supramolecular interactions as supramolecular anchor, leading to a 2D 4-fluorophenethylammonium lead iodide ((F-PEA)_2_PbI_4_) large dimensional size. The X-ray detector using the 1.5 mm thick 2D (F-PEA)_2_PbI_4_ single crystal as the photoconductor exhibited a sensitivity of 3402 μC Gy_air_^−1^ cm^−2^ to 120 keVp hard X-rays at 200 V bias, and the lowest detectable X-ray dose rate was as low as 23 nGy_air_ s^−1^. The 2D (F-PEA)_2_PbI_4_ also performed excellent operation stability during storage for over one month under ambient condition, demonstrating promising applications in hard X-ray detection [55].

The quantum well structure in 2D OIHP exhibit X-ray anisotropy detection properties, which is attributed to the mismatch of dielectric constant between the well and the barrier. Xu et al. produced 2D (BA)_2_CsPb_2_Br_7_ and (i-BA)_2_CsPb_2_Br_7_ crystals by inserting butylamine (BA) and isobutylamine (i-BA) into CsPbBr_3_ using the temperature cooling method and systematically studied their anisotropic X-rays optoelectronic properties. The quantum wells of 2D (BA)_2_CsPb_2_Br_7_ and (i-BA)_2_CsPb_2_Br_7_ grew along the ab plane while being perpendicular to the c direction. The device based on the (BA)_2_CsPb_2_Br_7_ crystal along the ab plane exhibited superior X-ray sensitivity of up to 13.26 mC Gyair^−1^ cm^−2^ at a relatively low electric field of 2.53 V mm^−1^ while displaying sensitivity lower than 20 μC Gy_air_^−1^ cm^−2^ along the c direction, even at a pretty high electric field of around 30 V mm^−1^ under the same irradiation of 40 kVp. By shortening the spacer cation from BA to i-BA, the degree of anisotropy in 2D perovskite crystals decreased, which resulted in lower ab plane X-ray sensitivity and higher c direction sensitivity compared to (BA)_2_CsPb_2_B_r7_-based devices [56]. The 2D OIHP-like (NH_4_)_3_Bi_2_I_9_ shows remarkable anisotropy of charge transport as well. The X-ray sensitivity of the parallel direction device was found to have a large value of 8.2 × 10^3^ μC Gy_air_^−1^ cm^−2^, while the sensitivity of the perpendicular direction device only revealed 803 μC Gy_air_^−1^ cm^−2^ under the irradiation of X-ray with a peak energy at 22 keV [57]. The anisotropy could effectively reduce horizontal charge migration between pixels and form nature arrays, which showed high resolution of X-ray imaging, e.g., a 34.3 mm × 34.3 mm × 13.1 mm (NH_4_)_3_Bi_2_I_9_ single-crystal-based device exhibited good spatial resolution of 4.22 lp mm^−1^ at parallel direction [58], which is higher than 3D MAPbI_3_-based X-ray detection (3.1 lp mm^−1^) [59]. In summary, X-ray detection along the growth direction of the quantum well is beneficial for improving image quality.

More X-ray optoelectronic properties of different 2D OIHPs can be found in Table 1, which shows a comprehensive comparison of recent progress in 2D OIHPs-based X-ray detectors.

There is no dispute that sensitivity is one of the most important parameters to study the performance of direct X-ray detectors. Unlike other X-ray photoconductors, such as a-Se, hybrid perovskites are seen as the candidate with the most potential for directly detecting both soft and hard X-rays. Thus, we found that the sensitivity of perovskite-based devices is often tested under different conditions. However, it would be difficult to compare the performance from different works, as seen in Table 1, because both the X-ray energy and bias have significant effects. In this review, we suggest that future publications should at least provide the detailed conditions of X-ray energy (average keV or kVp) and electric field intensity. If possible, it would be better to use a standard energy and bias scale for measuring perovskite-based devices.

In any case, the 2D OIHP currently studied in the field of X-ray detection is mostly single crystal, as shown in Table 1. The growth time of large single crystals is long, and the size is difficult to control. In situ growth of a vertical oriented 2D OIHP film on the substrate can obtain a functional layer with a controllable size. A study on a device obtained by in situ growth process exhibited low X-ray sensitivity as the thickness of the film was only 500 nm and the X-ray absorption was weak [62]. Therefore, adjusting the nucleation, growth, and interlayer force of 2D OIHP by reactants to construct a thick film with in situ vertical orientation as mentioned above is key to the application of 2D OIHP in X-ray detectors.

## 5. Conclusions and Outlook

Carrier transport in 2D OIHP is anisotropic, which is attributed to the confinement effect of the quantum well. Thus, high X-ray sensitivity can be usually obtained along the growth direction of the octahedron. In order to improve the application of 2D OIHP in X-ray detection, we need to build a thick 2D OIHP film that orientates perpendicular to the electrode to improve the collection efficiency of carriers. The vertical orientation of 2D OIHP is mainly achieved by introducing a reactant that can easily coordinate with the cation centered at the octahedrons to form intermediates and inhibit the rapid aggregation of the octahedrons. Speeding up the volatilization rate of solvents or additives to accelerate the conversion of intermediates to 2D OHIP is also a common strategy to optimize orientation. In addition, the orientation of 2D OIHP can be improved by adjusting the force between the spacers and the reaction system. The resulting device is expected to become a key component of the next generation of X-ray detectors.

In order to further increase the thickness of vertically oriented film, it is necessary to find a reagent that easily coordinates with cations in the octahedron and at the same time volatilizes with the solvent under milder conditions during the crystallization process, thereby enhancing the out-of-plane orientation, forming a pure-phase quantum well film, and enlarging the crystalline grains to improve the X-ray absorption and charge collection efficiency of 2D OIHP.

## Figures and Tables

**Table 1:** The optoelectronic properties of different 2D OIHPs.

Chemical Formula	2D Type	Device Structure	Ab Plane Sensitivity (μC Gy_air_^−1^ cm^−2^)	Limit of Detection (nGy_air_ s^−1^)	μτ (cm^2^V^−1^)	Response Time
(BDA)PbI_4_ SC [54]	DJ	Ag/OIHP/Ag	242 (40 kVp, 310 V/mm)	430 (40 kVp, 310 V/mm)	N/A	τ_rise_ = 7.3 ms, τ_fall_ = 22.5 ms
BA_2_CsAgBiBr_7_ SC [12]	RP	Au/OIHP/Au	4.2 (70 keV *, 10 V)	N/A	1.21 × 10^−3^	N/A
BA_2_EA_2_Pb_3_Br_10_ SC [60]	RP	Au/OIHP/Au	6800 (70 keV *, 5 V/mm)	5500	1.0 × 10^−2^	N/A
(CPA)_4_AgBiBr_8_ SC [61]	RP	Au/OIHP/Au	0.8 (70 keV *, 10 V)	N/A	1.0 × 10^−3^	N/A
(BA)_2_(MA)_2_Pb_3_I_10_ film [62]	RP	p-i-n ITO/PTAA/OIHP/C_60_/Au	~13 (10.91 average keV, 0 V/mm)	N/A	N/A	τ_rise_ < 500 ns, τ_fall =_ 20–60 μs
(F-PEA)_2_PbI_4_ SC [56]	RP	Au/OIHP/C_60_/BCP/Cr	3402 (120 keVp, 133 V/mm)	23 (120 keVp, 133 V/mm)	5.1 × 10^−4^	0.8 μs
(BA)_2_CsPb_2_Br_7_ SC [57]	RP	Au/OIHP/Au	13,260 (40 kVp, 2.53 V/mm)	72.5 (40 kVp, 2.53 V/mm)	N/A	N/A
(DFPIP)_4_AgBiI_8_ SC [63]	RP	Au/OIHP	188 (40 kVp, 50 V)	3130 (40 kVp, 50 V)	1.1 × 10^−5^	N/A

N/A means that it is not mentioned in the reference, SC refers to single crystal, * indicates the highest energy instead of the average energy of the X-ray source.
